# Meta-analysis of the prevalence of depression among breast cancer survivors in Iran: an urgent need for community supportive care programs

**DOI:** 10.4178/epih.e2019030

**Published:** 2019-07-04

**Authors:** Hassan Ahmadi Gharaei, Mostafa Dianatinasab, Seyyed Mostafa Kouhestani, Mohammad Fararouei, Hossein Moameri, Reza Pakzad, Reza Ghaiasvand

**Affiliations:** 1 Department of Epidemiology, Faculty of Public Health, Tehran University of Medical Sciences, Tehran, Iran; 2 Department of Epidemiology, Center for Health Related Social and Behavioral Sciences Research, Shahroud University of Medical Sciences, Shahroud, Iran; 3 Department of Health Management and Economics, School of Public Health, Tehran University of Medical Sciences, Tehran, Iran; 4 Department of Epidemiology, Faculty of Public Health, Shiraz University of Medical Sciences, Shiraz, Iran; 5 Noor Research Center for Ophthalmic Epidemiology, Noor Eye Hospital, Tehran, Iran; 6 Faculty of Health, Ilam University of Medical Sciences, Ilam, Iran; 7 Oslo Centre for Biostatistics and Epidemiology, Department of Biostatistics, Institute of Basic Medical Sciences, University of Oslo, Oslo, Norway

**Keywords:** Breast carcinoma, Depression, Supportive care, Quality of life, Prevalence, Meta-analysis

## Abstract

**OBJECTIVES:**

Depression, which is the most common comorbidity in breast cancer (BC) patients, has adverse effects on patients’ quality of life, disease progress, and survival.

**METHODS:**

The protocol of this study was registered in PROSPERO (registration No. CRD42019121494). We electronically searched published studies through January 2019 with the aim of finding articles that investigated the prevalence of depression among BC survivors. Web of Science, Scopus, PubMed/MEDLINE, Science Direct, and Google Scholar were searched to obtain relevant published studies. This review included 14 cross-sectional and 4 cohort studies published from 2000 to 2018. We used a random-effects model to conduct the meta-analysis and generated a summary estimate for the pooled prevalence with 95% confidence intervals (CIs). A subgroup analysis was also conducted based on the depression assessment tool used and the study design.

**RESULTS:**

The total sample size of the studies contained 2,799 women with BC, including 1,228 women who were diagnosed with depression. The pooled prevalence of depression among Iranian women with BC was 46.83% (95% CI, 33.77 to 59.88) with significant heterogeneity (I^2^ =98.5%; p<0.001). The prevalence of depression ranged from 14.00% (95% CI, 4.91 to 23.09) to 95.90% (95% CI, 91.97 to 99.83). The results of the subgroup analyses suggested that the depression assessment tool, year of publication, and study design were sources of heterogeneity.

**CONCLUSIONS:**

Our findings indicate a high prevalence of depression among BC patients, underscoring the urgent need for clinicians and health authorities to provide well-defined social and psychological supportive care programs for these patients.

## INTRODUCTION

It is estimated that about 2.1 million newly diagnosed cases of breast cancer (BC) occurred in 2018 worldwide, accounting for almost 1 in 4 of all cases of cancer among women. BC is the most frequently diagnosed cancer in most countries (154 of 185), and is the leading cause of death due to cancer in over 100 countries [1]. In Iran, BC is the most common cancer in women; it has a high mortality rate [2], and the mean age at diagnosis is significantly lower in Iranian women than in their Western counterparts [3,4]. As a result, it is among the most important challenges in public health [5]. However, because of newly advanced diagnostic and treatment strategies [6,7], the survival rate of patients has increased significantly. As a result, about 89% of patients now survive for at least 5 years after diagnosis [8]. Thus, focus has now turned toward enhancing patients’ quality of life (QoL), as patients often experience persistent aversive symptoms such as fatigue, cognitive difficulties, and mental problems [9]. The increase in patients’ life expectancy is accompanied by a longer exposure to negative psychological impacts of BC [9].

When diagnosed with cancer, patients are exposed to an instigator of many fears, including fears of death, separation, and isolation from their loved ones, as well as fear of deterioration and pain. The most common psychological disorders among cancer patients are mood disorders, anxiety, depression, and sexual dysfunction. For example, the period from the diagnosis of BC to months following primary therapy is long and highly stressful, which causes psychological instability and depression [5]. Depression, which is the most common comorbidities among BC survivors, diminishes QoL and may lead to an increase in the cost of healthcare [10]. An international study showed an increasing prevalence of depression among women with BC [11]. Therefore, promoting mental and physical health is becoming an important aspect of all related healthcare programs [6]. Evidence has suggested that depression may affect the progression and survival of cancer [12,13].

Helping patients with cancer achieve the best possible QoL is a shared goal among medical and healthcare communities [14]. Hence, with regard to the increasing number of BC patients and the increasing prevalence of psychological disorders among BC survivors, it is very important to understand the prevalence of depression and its impact on patients’ health status. Robust findings on this topic underscore the urgent need for clinicians and health authorities to provide well-defined social and psychological supportive care programs for BC survivors.

## MATERIALS AND METHODS

The present systematic review and meta-analysis was prepared according to the PRISMA (Preferred Reporting Items for Systematic Reviews and Meta-Analyses) statement [15].

### Protocol and registration

The purpose of the present study was to determine the prevalence of depression among women with BC in Iran. The study protocol was registered in PROSPERO, an international prospective register of systematic reviews, under the registration No. CRD42019121494.

### Search strategy and selection criteria

A comprehensive search was performed with the aim of finding any cross-sectional or cohort studies that investigated the prevalence of depression among patients with BC in Iran. Accordingly, the following sources were searched: Web of Science, Scopus, PubMed/MEDLINE, Science Direct, Google Scholar, Magiran, Scientific Information Database, IranMedex, and Medlib. Four sets of related MeSH and non-MeSH terms in titles, abstracts, or keywords were used: (1) “breast cancer” OR “breast carcinoma” OR “breast neoplasm”, (2) “depression” and (3) “prevalence” OR “occurrence” and (4) “Iran” were searched for articles published from February 2000 until January 2019.

The search strategy was performed using Boolean operators (AND, OR). Two authors independently reviewed the articles (HAG and SMK) and discrepancies were resolved by discussing with the third author (MD). We also manually reviewed the reference lists of related articles for other possibly relevant articles that were not found through the electronic search strategy.

### Inclusion criteria

Prospective cohort studies and cross-sectional studies that investigated the prevalence of depression among Iranian BC patients were included, encompassing both studies on newly diagnosed BC cases (incident cases) and prevalent cases of BC.

### Exclusion criteria

Prospective cohort studies and cross-sectional studies that did not report the prevalence of depression among Iranian BC patients, reported the prevalence of depression among patients with other types of cancer (not relevant or modified data), reported the prevalence of depression among other comorbidities of BC patients, or focused on BC recurrence were not included in our systematic review and meta-analysis. Studies in review design or letter to the editors (not a research article) were also excluded.

### Data extraction

Two authors (HAG and SMK) independently extracted the following data for each study: authors’ name, publication year, setting, sample size, numbers of patients who suffered from depression, prevalence of depression, age, the tool used, marital status, and study design. If the full text of any article was unavailable or if key information was missing from the reported data, up to 2 attempts to contact the authors were made at 1-week intervals, and we also sent an email to the publishers (e.g., the Elsevier and Wiley online library).

### Quality of assessment

An 8-item checklist for the critical appraisal of studies of the prevalence/incidence of health problems [16] was used to examine the quality of eligible studies by 2 independent investigators (HAG and SMK). The checklist contained items assessing the following criteria: (1) whether a random sample or a whole population was used, (2) the use of an unbiased sampling frame, (3) adequacy of the sample size, (4) the use of standard measures, (5) whether outcome measurements were made by unbiased assessors, (6) adequacy of the response rate, (7) confidence intervals (CIs) and subgroup analyses, and (8) whether the study subjects were described. Each item was scored as 1 if a study met the criterion, and the scores were summed up. The range of the total score was from 0 (lowest possible quality) to 8 (highest possible quality). The quality assessment results were also checked by a third investigator (MD).

### Statistical analysis

A random-effects model was used to investigate the pooled prevalence of depression with 95% CIs among women with BC. The I^2^ test was used to evaluate the heterogeneity of studies [17]. Subgroup analyses were performed based on the study design, year of publication, the depression assessment tool used and quality of studies. To study the heterogeneity of sources, meta-regression was used based on the design and quality of studies, sample size, age of women, the tool used to assess depression, and publication year. The Egger test [18] and funnel plot method [19] were used to test the severity of publication bias. Moreover, a sensitivity analysis was conducted to assess the stability of the results. Stata version 14 (StataCorp., College Station, TX, USA) was used for the statistical analysis [20].

### Ethics statement

Ethical approval was obtained from Tehran University of Medical Sciences. This was a review study, so consent to participate was unnecessary.

## RESULTS

### Included studies

**Figure 1.**
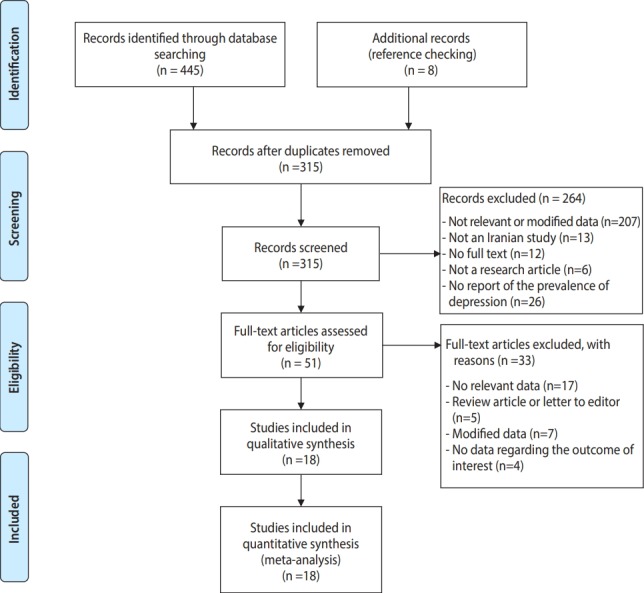
Flowchart of study inclusion and exclusion according to the PRISMA (Preferred Reporting Items for Systematic Reviews and MetaAnalyses) statement.

As described in Figure 1, which shows the PRISMA flow chart, a total of 453 records were obtained by electronic and manual searching. After duplicate references were removed, 315 records remained for further assessment. We excluded 264 studies after screening titles and/or abstracts due to the following reasons: not being relevant (n=207), not having been conducted in Iran (n=13), not having the full text available (n=12), being a review/letter to the editor or poster presentation (n=6), and not reporting the prevalence of depression (n=26). Therefore, 51 studies remained to be carefully checked by examining the full texts, of which 33 articles were excluded for the following reasons: not containing relevant data (n=17), not being an original article (i.e., being a review article or letter to the editor, n=5), using modified data (n=7), or not reporting the outcome of interest (n=4). In total, 18 articles met the criteria for qualitative synthesis in the systematic review and the quantitative meta-analysis. The characteristics of the studies included in this review are presented in Table 1. The study design was cross-sectional (n=14) [2,4,21-32] or cohort (n=4) [33-36].

Overall, the included studies contained 2,799 women with histologically confirmed BC (mean sample size, 155.50±87.35 patients). Of those patients, 1,228 (43.87%) were diagnosed with depression, and the mean age of the patients was 47.94 years (standard deviation, 3.76).

Six different methods were used to diagnose depression in the included studies. Seven studies used the Beck Depression Inventory (BDI) [21,23-25,29-31], 2 studies used the Depression, Anxiety, and Stress Scale–42 (DASS-42) [27,32], 6 studies used the Hospital Anxiety and Depression Scale (HADS) [22,26,28,33,34, 36], 1 study used the Diagnostic and Statistical Manual of Mental Disorders, 4th edition (DSM-IV) [35], 1 study used the Distress Thermometer (DT) [2], and 1 study used the Center for Epidemiological Studies Depression Scale (CES-D) [4].

### Assessment of methodological quality

According to the critical appraisal of studies of prevalence/incidence of a health problem checklist [16], 2 studies were classified as high-quality studies (score ≥7) [33,36], 6 as medium-quality studies (score between 4 and 6) [21,22,24,28,30,35] and 10 as low-quality studies (score <4) [2,4,23,25-27,29,30,32,34] (Supplementary Material 1).

### Pooled prevalence of depression

The highest prevalence of depression among women with BC was 95.90% (95% CI, 91.97 to 99.83) which was reported by Shakeri et al. [30] in Kermanshah, and the lowest prevalence was 14.00% (95% CI, 4.91 to 23.09) reported by Montazeri et al. [34] in Tehran. Using a random-effects model, we found that the pooled prevalence of depression among women with BC was 46.83% (95% CI, 33.77 to 59.88). The results of the included studies showed highly significant heterogeneity (I^2^=98.5%; p<0.001).

### Meta-regression

**Figure 2.**
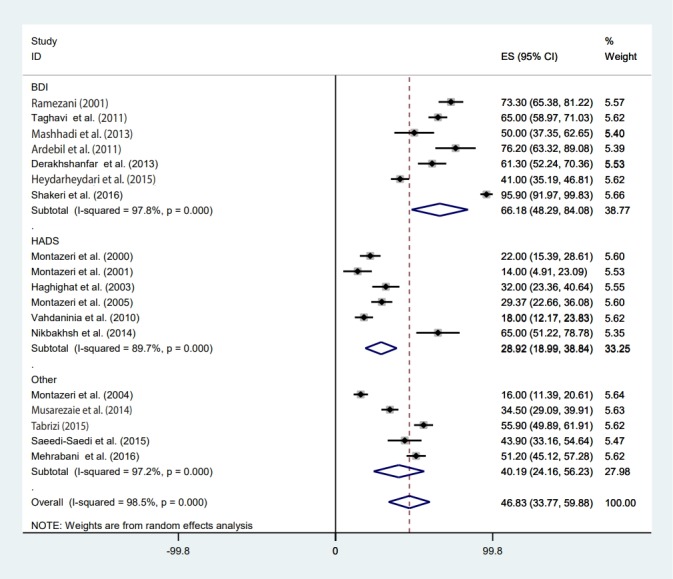
Forest plot of all 18 studies included in the meta-analysis based on the depression assessment tool used. ES, effect size (prevalence); CI, confidence interval; BDI, Beck Depression Inventory; HADS, Hospital Anxiety and Depression Scale.

**Figure 3.**
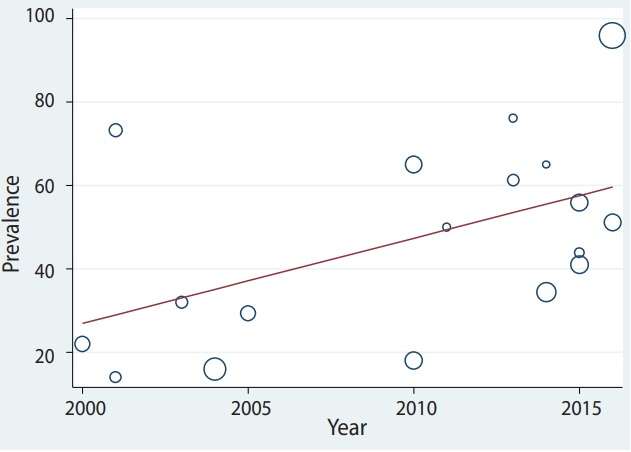
Prevalence of depression among Iranian breast cancer, showing an increase in prevalence from 2000 to 2018.

**Figure 4.**
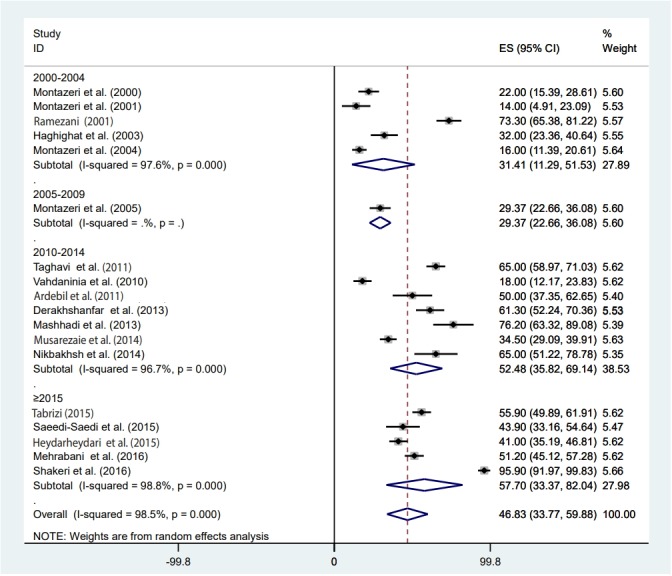
Prevalence of depression among Iranian breast cancer patients based on the year of publication. ES, effect size (prevalence); CI, confidence interval.

Meta-regression was conducted according to study design, the measurement method, participants’ age, the article’s publication year, sample size, and studies’ quality score (Table 2). Heterogeneity was found for study design, the measurement method, and the publication year of the article. Based on the results of meta-regression, the prevalence of depression had a significant association with study design (β=-37.72; p=0.001). The prevalence of depression in the prospective studies was 37% lower than in cross-sectional studies (Table 2). Furthermore, the prevalence of depression had a significant association with the measurement method (β=-14.25; p=0.03) (Table 2). The prevalence of depression measured by the BDI was 66.18% (95% CI, 48.29 to 84.08), that measured by the HADS was 28.92% (95% CI, 18.99 to 38.84) and that measured by other methods was 40.19% (95% CI, 24.16 to 56.23) (Figure 2). We found a significant upward trend in the prevalence of depression among women with BC in recent years. As shown in Figures 3 and 4, the prevalence of depression during 2000-2004 was 31.41% (95% CI, 11.29 to 51.53), during 2005-2009 it was 29.37% (95% CI, 22.66 to 36.08), between 2010 and 2014 it was 52.48% (95% CI, 35.82 to 69.14), and in 2015 or later it was 57.70% (95% CI, 33.37 to 82.04) (β=9.42; p=0.04).

### Publication bias and sensitivity analysis

**Figure 5.**
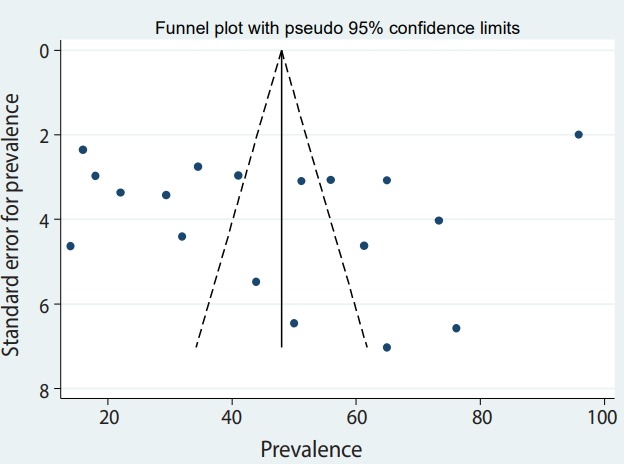
Funnel plot of the studies included in the meta-analysis.

**Figure 6.**
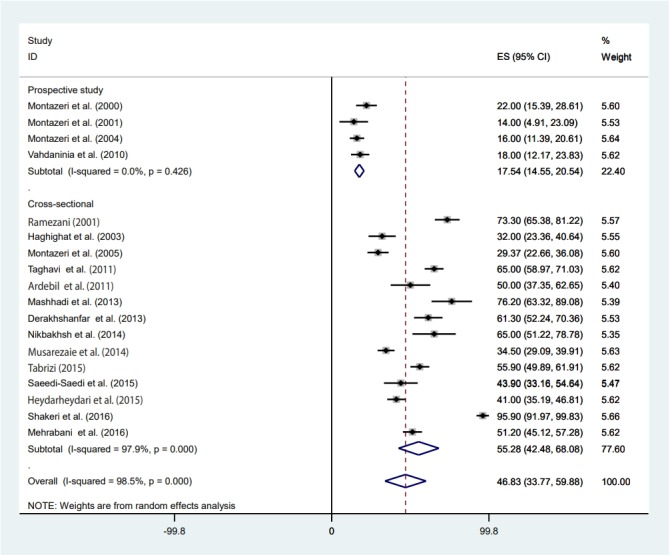
Forest plot of all studies based on study design. ES, effect size (prevalence); CI, confidence interval.

The Egger test and funnel plot method were used to measure publication bias. As presented in Figure 5, no significant publication bias was found (t=-0.57; p=0.57). The sensitivity analysis is shown in Table 3. The results of the subgroup analysis by study design and tool for depression assessment are shown in Figures 2 and 6, respectively.

### Estimate of pooled prevalence in the subgroup analysis based on the result of meta-regression

The pooled depression prevalence measured by the BDI, HADS, and other methods was 66.18% (95% CI, 48.29 to 84.08), 28.92% (95% CI, 18.99 to 38.84), and 40.19% (95% CI, 24.16 to 56.23), respectively (Figure 2). Moreover, the prevalence of depression reported by cross-sectional and prospective studies was 55.28% (95% CI, 42.48 to 68.08) and 17.54% (95% CI, 14.55 to 20.54), respectively (Figure 6). The depression prevalence based on year of publication was 22.00% (95% CI, 15.39 to 28.61) and 73.61% (95% CI, 29.81 to 100.00) in 2000 and 2016, respectively (Figure 4).

## DISCUSSION

Depression is a common condition among BC patients, but it is often unrecognized and therefore untreated. The condition intensifies physical symptoms, resulting in additive functional impairment and poor adherence to treatment. As a result, depression in BC patients is responsible for significant deterioration in QoL [9].

The current systematic review and meta-analysis was conducted to investigate the prevalence of depression among Iranian women diagnosed with BC. The high prevalence (46.8%) of depression observed in this study among BC survivors is worrying, and it calls for immediate attention. We found that the lowest and highest reported prevalence rates were 14.0% in Tehran [34] and 95.9% in Kermanshah [30], respectively. Our results suggest that the prevalence of depression among Iranian BC patients is higher than that of postpartum depression in Iran, depression among Iranian infertile couples, or depression among Iranian adolescents [37-39]. According to the results of a systematic review, the global prevalence of depression among women with BC ranged from 1% in London to 56% in USA [40]. Furthermore, another study reported that the prevalence of depression among Asian women with BC was between 12.5% and 31.0% [40]. Our study showed that the prevalence of depression among Iranian women with BC is higher than rates that have been reported globally [41,42]. A study conducted in Turkey reported the prevalence of depression among BC patients ranged from 27.7% (moderate depression) to 19.5% (major depression) [41]. Similarly, a previous systematic review on prevalence of depression and anxiety after BC treatment showed a lower rate of depression among women with BC (between 9.4% and 66.1%) [42]. The observed differences in the prevalence of depression among women from different countries are likely due to differences in cultural, behavioral, and demographic characteristics, including the economic status of the population [43-45], age (Iranian patients are younger) [36], social support [46], education [47], and marital status [48]. In addition, discrepancies in cancer stage and illness duration, differences in the methods used to measure depression, and variation in sample size and methods of sampling may have led to different prevalence rates.

The results of this study showed that the prevalence of depression increased from 2000 to 2017; the prevalence of depression in 2000-2004 years was 31.4%, and after 2015 it was around 57.7%. Many factors may have an effect on the prevalence of depression among BC patients, including increases in the prevalence and incidence of BC, increases in the survival rate of patients, improvements in diagnostic methods, and the launch of a cancer registration system.

It seems that in Iranian society, for cultural reasons, women are placed under additional pressure following the diagnosis and treatment of BC. As a result, while suffering from physical complications of the disease and its treatment, BC patients suffer from stress and emotional problems [49]. The higher prevalence of depression among BC survivors in Iran can be explained as the result of lack of support from society and patients’ families. As BC and its treatment strongly influence the daily activity and sexual performance of survivors at sexually active ages, younger patients are especially prone to depression. It has been suggested that the risk of sexual dysfunction is higher among younger women due to the dependence of their sexual desire on their body image and a reduction in their self-esteem after BC treatment (i.e., mastectomy) [50]. Furthermore, psychological problems and sexual dysfunction after BC treatment have been reported in the majority of patients [51,52]. For example, a study of Iranian patients suggested that after treatment (i.e., mastectomy), women had negative feelings about their bodies [49]. It has also been suggested that when Iranian women with BC are about to engage in sexual intercourse, they face anxiety, shame, and negative feelings about their bodies [49].

The opinion of relatives, especially the husband’s relatives, about the patient is another source of stress. Emotional support from their husbands is therefore fundamentally important for patients during the treatment of BC. It has been shown that society’s and relatives’ attitude toward women who have lost their feminine organs have significant effects on their feelings, potentially resulting in depression and anxiety [52]. This issue is mainly due to the cultural context of society, and to some extent the religious beliefs of a community toward women’s responsibility as wives to build a satisfactory sexual relationship with their husbands. When facing challenges in fulfilling these expectations, patients may feel incompetent and sense that they are subject to tremendous pressure [49,51]. These factors, along with the physical complications of the disease and its treatments, may be unbearable to patients who need social and emotional support. This lack of support makes patients prone to depression and reduces their QoL [49].

Another important factor that may have a significant effect on depression among patients with BC is the late diagnosis of BC, as around 70% of Iranian patients with BC are diagnosed at an advanced stage [3], which is again due to low levels of knowledge and access to medical services and cultural barriers [53]. As a consequence of late diagnosis, patients face more aggressive tumors, require more aggressive treatment (i.e., mastectomy), and have a poorer prognosis. It has been suggested that patients who receive aggressive treatment or surgery are more prone to psychological problems, including depression, as breasts are a symbol of femininity and their partial or total removal can cause irreversible damage to patients’ feelings and emotional state [54].

Along with the previously discussed issues, the observed difference in the prevalence of depression reported by previous studies can also be explained in terms of the studies’ design. In this review study, the prevalence of depression reported in cross-sectional studies was much higher (55.3%) than that reported in prospective studies (17.5%). In line with this result, another review article reported that the prevalence of depression in cross-sectional studies was higher than in prospective studies [40]. A possible reason for this is that cross-sectional studies usually include both new and old cases of depression. In addition, the tools used by the studies included in this systematic review were rather heterogeneous. In studies that used the BDI scale, the prevalence of depression among BC patient was much higher (66.2%) than that reported in studies using the HADS (28.9%), and studies utilizing other scales (DSM-IV, DASS-42, CES-D, and the DT) showed an intermediate prevalence of depression (around 40%). The BDI seems to be a valid screening tool for depression in advanced cancer patients [55], although the HADS is also a useful screening tool for depression in cancer patients [56]. However, it seems that the differences in the results of the BDI and HADS should be acknowledged, meaning that results should be compared with caution. A possible reason for discrepancies in the results of different depression scales was provided by Cusin et al. [57]. They stated that only the HADS is suitable for measuring depression in those with a serious medical condition; other measures might overestimate the scale of depression, since they do not exclude somatic factors [57]. However, there is no consensus on this issue, as Hann et al. [58] evaluated the CES-D in BC patients and concluded that the scale was a valid and reliable measure for diagnosing depression in BC survivors. In a similar vein, Osborne et al. [59] investigated the validity of the HADS in BC survivors, and only found minor psychometric problems.

The results of the current study revealed a high prevalence of depression among women with BC. Suffering from depression can exacerbate the prognosis of the disease and reduce patients’ survival [60]. According to the results of this study, there was no relationship between prevalence of depression and the age of women with BC. Our finding is consistent with the results of a study by Vin-Raviv et al. [61] in this regard. However, other studies have revealed a direct association between the prevalence of depression and age [62].

The present study has some limitations that must be pointed out. Firstly, data on some important factors, including the stage of BC and the years since diagnosis, were not available, making it impossible to conduct a subgroup analysis of whether the prevalence of depression differed according to those factors. Secondly, Iran is a country with considerable ethnic diversity, which may affect the prevalence of depression in different areas of the country. However, it was not possible for us to determine the possible role of ethnicity in these findings. In contrast, strengths of this study include the fact that national and international databases were searched in both Farsi (Persian) and English to obtain comprehensive results, and that we excluded studies on BC recurrence because this may be a source of bias.

## CONCLUSION

The results of this systematic review revealed a remarkably high prevalence of depression among women with BC. Early diagnosis, social support, and emotional support from family members can help in the management of depression, with positive impacts on QoL. These results should be carefully considered by physicians, healthcare providers, and the patient’s family when a woman is diagnosed with BC.

## Figures and Tables

**Table 1.** The main Characteristics of the studies included in the present systematic review and Meta-analysis

Study	Province	Design	Age, Mean±SD	Marital status, %			Study sample size, n	No. of cases of depression, n	Depression prevalence, %	95% CI		Tools
				Single	Married	Widowed				LL	UL	
Shakeri et al., 2016 [30]	Kermanshah	Cross-sectional	47.60±14.05	-	-	-	98	94	95.90	91.97	99.83	BDI
Mehrabani et al., 2016 [32]	Neyshabur	Cross-sectional	55.91±14.55	-	-	-	260	133	51.28	45.12	57.28	DASS-42
Heydarheydari et al., 2015 [23]	Kermanshah	Cross-sectional	46.27±7.21	7.3	81.8	10.9	275	115	41.00	35.19	46.81	BDI
Tabrizi, 2015 [4]	Urmia	Cross-sectional	47.90±11.40	9.0	91.0	-	262	146	55.90	49.89	61.91	CES-D
Saeedi-Saedi et al., 2015 [2]	Rasht	Cross-sectional	50.10±10.96	4.9	85.4	9.7	82	36	43.90	33.16	54.64	DT
Nikbakhsh et al., 2014 [28]	Babol	Cross-sectional	59.04±14.30	2.0	97.3	0.7	46	30	65.00	51.22	78.78	HADS
Musarezaie et al., 2014 [27]	Isfahan	Cross-sectional	46.10±14.03	8.4	76.1	15.5	297	102	34.50	29.09	39.91	DASS-42
Derakhshanfar et al., 2013 [25]	Hamedan	Cross-sectional	47.05±11.68	5.4	84.7	9.9	111	68	61.30	52.24	70.36	BDI
Ardebil et al., 2011 [21]	Tehran	Cross-sectional	43.81±47.12	-	-	-	60	30	50.00	37.35	62.65	BDI
Mashhadi et al., 2013 [24]	Zahedan	Cross-sectional	45.00±8.50	-	-	-	42	32	76.20	63.32	89.08	BDI
Taghavi et al., 2011 [31]	Isfahan	Cross-sectional	46.43±ND	9.2	72.5	18.3	240	156	65.00	58.97	71.03	BDI
Vahdaninia et al., 2010 [36]	Tehran	Prospective	47.20±13.50	9.0	69.4	21.6	167	30	18.00	12.17	23.83	HADS
Montazeri et al., 2005 [26]	Tehran	Cross-sectional	48.20±14.90	11.3	78.5	10.1	177	52	29.37	22.66	36.08	HADS
Montazeri et al., 2004 [35]	Tehran	Prospective	46.60±11.20	4.0	84.0	12.0	243	39	16.00	11.39	20.61	DSM-IV
Haghighat et al., 2003 [22]	Tehran	Cross-sectional	45.70±11.10	7.0	85.0	7.0	112	35	32.00	23.36	40.64	HADS
Ramezani, 2001 [29]	Kerman	Cross-sectional	47.53±10.57	3.3	96.7	-	120	88	73.30	65.38	81.22	CES-D
Montazeri et al., 2001 [34]	Tehran	Prospective	45.40±9.20	18.0	82.0	-	56	8	14.00	4.91	23.09	HADS
Montazeri et al., 2000 [33]	Tehran	Prospective	47.20±13.50	9.0	70.0	21.0	151	34	22.00	15.39	28.61	HADS

SD, standard deviation; CI, confidence interval; LL, lower limit; UL, upper limit; ND, no data; BDI, Beck Depression Inventory; DASS-42, Depression, Anxiety and Stress Scale-42; CES-D, Center for Epidemiological Studies Depression Scale; DT, Distress Thermometer; HADS, Hospital Anxiety and Depression Scale; DSM-IV, Diagnostic and Statistical Manual of Mental Disorders, 4th eds.

**Table 2.** Heterogeneity among the selected studies based on meta-regression

Variables	Beta	95% CI		p-value
		LL	UL	
Quality of study	6.60	-10.51	23.72	0.42
Design	-37.72	-58.47	-16.97	0.001
Age	0.19	-1.21	1.59	0.77
Year	9.42	4.28	2.20	0.04
Sample size	-0.06	-0.02	0.07	0.32
Tools	-14.25	-27.02	-1.47	0.03

CI, confidence interval; LL, lower limit; UL, upper limit.

**Table 3.** Sensitivity analysis of studies included in the meta-analysis

Study	Estimate	95% CI	
		LL	UL
Shakeri et al., 2016 [30]	43.73	34.32	53.14
Mehrabani et al., 2016 [32]	46.57	32.63	60.13
Heydarheydari et al., 2015 [23]	47.18	33.22	61.13
Tabrizi, 2015 [4]	46.29	32.38	60.20
Saeedi-Saedi et al., 2015 [2]	47.00	33.40	60.59
Musarezaie et al., 2014 [27]	47.56	33.65	61.48
Nikbakhsh et al., 2014 [28]	45.80	32.31	59.28
Derakhshanfar et al., 2013 [25]	45.98	32.37	59.59
Ardebil et al., 2011 [21]	46.64	33.10	60.19
Mashhadi et al., 2013 [24]	45.15	31.72	58.58
Taghavi et al., 2011 [31]	45.75	31.99	59.50
Vahdaninia et al., 2010 [36]	48.54	35.22	61.85
Montazeri et al., 2005 [26]	47.86	34.19	61.53
Montazeri et al., 2004 [35]	48.66	35.70	61.62
Haghighat et al., 2003 [22]	47.70	34.09	61.30
Ramezani, 2001 [29]	45.26	31.77	58.76
Montazeri et al., 2001 [34]	48.74	35.41	62.08
Montazeri et al., 2000 [33]	48.30	34.81	61.79
Combined	46.82	33.77	62.08

CI, confidence interval; LL, lower limit; UL, upper limit.
