# Supplementary material for: Meta-analysis of the prevalence of depression among breast cancer survivors in Iran: an urgent need for community supportive care programs
Source: Epidemiol Health. 2019 Jul 4;41:e2019030. doi: 10.4178/epih.e2019030 (PMC6791826; doi:10.4178/epih.e2019030)
Supplement: Supplementary file 1 [file epih-41-e2019030-supplementary1.pdf]

Volume: 41, Article ID: e2019030

https://doi.org/10.4178/epih.e2019030

Supplementary Material 1. Results of risk assessment bias using Guidelines for critically appraising studies of prevalence or incidence of a health problem

Study	Are the study methods valid?						What is the interpretation of the results?	What is the applicability of the results?	score
	a) Are the study design and sampling method appropriate for the research question?	b) Is the sampling frame appropriate?	c) Is the sample size adequate?	d) Are objective, suitable and standard criteria used for measurement of the health outcome?	e) Is the health outcome measured in an unbiased fashion?	f) Is the response rate adequate? Are the refusers described?	Are the estimates of prevalence or incidence given with confidence interval and in detail by subgroup, if appropriate?	Are the study subjects and the setting described in detail and similar to those of interest to you?	
Shakeri 2016 (22)	*	*		*	*			*	5
Mehrabani 2016 (27)				*	*		*	*	4

Volume: 41, Article ID: e2019030

https://doi.org/10.4178/epih.e2019030

Ramazani 2015 (20)				*			*	*	4
Heidar-heidary 2015 (30)				*	*		*	*	4
Moghaddam Tabrizi 2015 (24)			*	*	*		*	*	5
Saeedi-Saedi 2015 (25)				*	*		*	*	4
Nikbakhsh 2014 (19)	*	*		*	*		*	*	6
Rezaei 2014 (2001)			*	*	*		*	*	5
Derakhshan far 2013 (27)				*	*		*	*	4
Didedar-ardebil 2013 (23)				*	*	*	*	*	5

Volume: 41, Article ID: e2019030

https://doi.org/10.4178/epih.e2019030

Mashhadi 2013 (29)			*	*	*		*	*	5
Taghavi 2010 (21)				*	*		*	*	4
Vahdaninia 2010 (31)	*	*	*	*	*	*	*	*	8
Montazeri 2005 (26)				*	*		*	*	4
Montazeri 2004 (33)	*		*	*	*		*	*	6
Haghighat 2003 (28)	*			*	*		*	*	5
Montazeri 2001 (34)				*	*	*		*	4
Montazeri 2000 (32)		*	*	*	*	*	*	*	8
